# Application of Open‐Source Digital Resources for 3D Visualization of Clustered Transcriptomic Data

**DOI:** 10.1111/ppl.70500

**Published:** 2025-09-17

**Authors:** Hunter F. Strickland, Andrew Shen, Anna‐Lisa Paul, Robert Ferl

**Affiliations:** ^1^ Plant Molecular and Cellular Biology Program University of Florida Gainesville Florida USA; ^2^ Department of Horticultural Sciences University of Florida Gainesville Florida USA; ^3^ Department of Digital Arts and Sciences University of Florida Gainesville Florida USA; ^4^ Interdisciplinary Center for Biotechnology Research University of Florida Gainesville Florida USA; ^5^ Office of Research University of Florida Gainesville Florida USA

## Abstract

As datasets grow in size with the increased accessibility of high‐throughput transcriptome sequencing, methods of dimensionality reduction have become invaluable for data analysis. The methods of dimensionality reduction, including t‐distributed stochastic neighbor embedding or Uniform Manifold Approximation and Projection, are utilized to create figures and projections of the high‐dimensional data into a set of lower dimensions, 2D or 3D, which are more well‐suited for human comprehension. These methods of dimensionality reduction have continually grown in popularity and widespread use. Despite this popularity, creating engaging and visually attractive features remains an issue for many users without significant coding experience. To remediate this issue, an HTML‐based digital resource was created that utilizes publicly available scripts from JsDelivr and GitHub, and Blender, an open‐source modeling software. We have generated two open‐source digital data visualization resources that can be applied to the transcriptomic data processed using the aforementioned methods of dimensionality reduction. The first, HTMLview, utilizes a provided HTML file template to create an interactive and engaging 3D model in digital space. The second method, Blenderview, utilizes the open‐source modeling software, Blender, to create and animate high‐quality models and videos of processed datapoints. The two methods were tested with transcriptomic data processed via dimensionality reduction algorithms. The methods provided create two distinct paths for researchers to better visualize, examine, and share their data, while also utilizing open‐source technologies that are readily available to most potential users.

## 1 Introduction

Visualization of large, complex datasets is crucial for both understanding biological research data and for the communication of results. This fact is becoming increasingly more relevant as the application of high‐throughput transcriptomic technologies generates extensive datasets that span multiple dimensions. These datasets often require methods of dimensionality reduction, like t‐distributed stochastic neighbor embedding (t‐SNE) (van der Maaten and Hinton 2008) or Uniform Manifold Approximation and Projection (UMAP) (Becht et al. 2019), to become more accessible to the human eye. Often, these dimensionality reduction techniques partially ease the difficulty of communicating the data from large transcriptomic datasets with various audiences through the production of 2D representations of the data.

**FIGURE 1:**
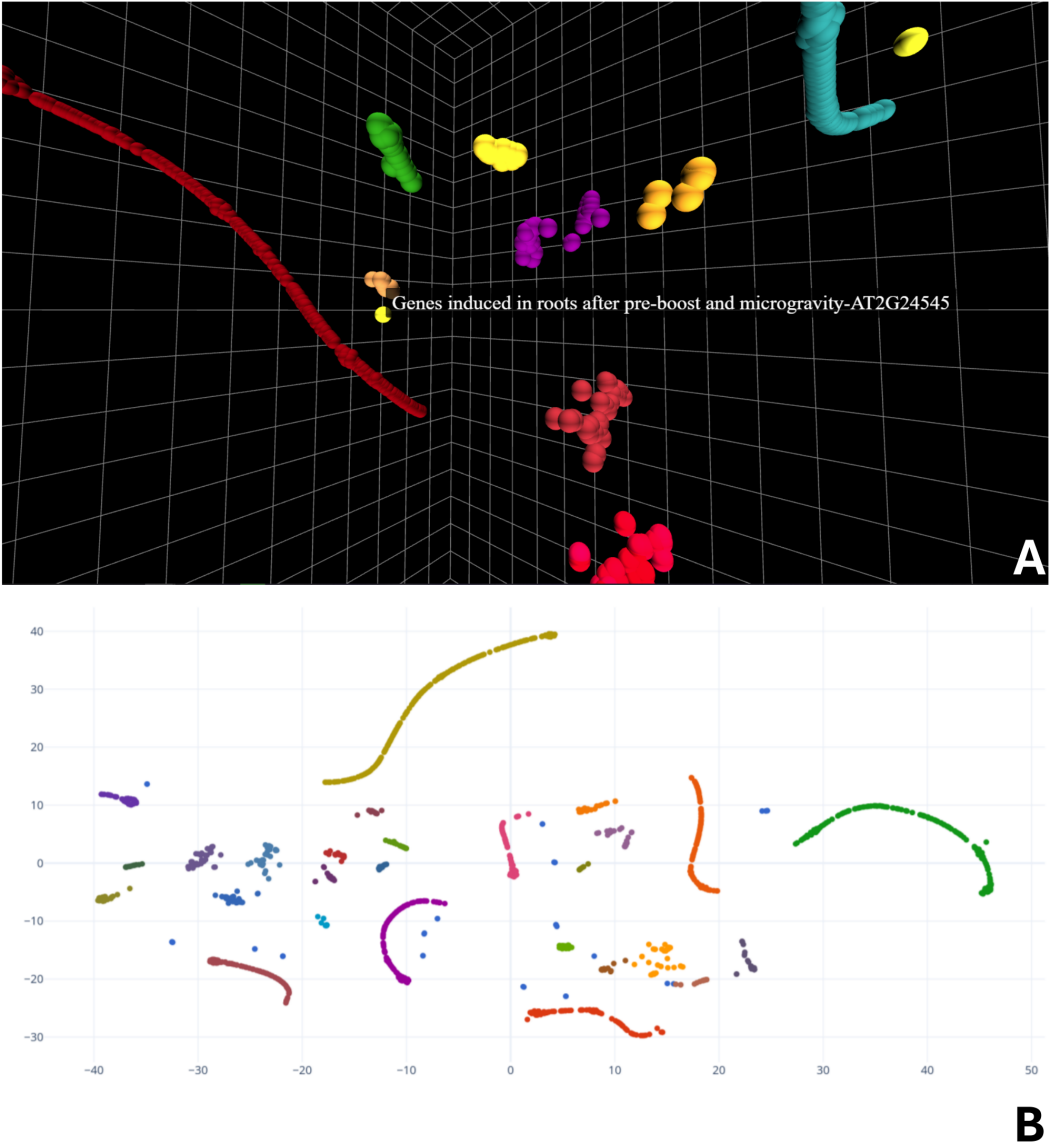
3D images are more immersive than typical 2D projections. An HTMLview projection is shown above, with each point representing a unique gene ID that has been clustered based on the significant Log2fold‐change values and then colored based on DBScan identified clusters (A). The same data was processed using Rtsne with a 2D projection, and the clusters identified with DBScan were colored with an alternative color scheme (B).

We suggest that explorable 3D models of these data greatly augment the visual interpretation of transcriptomic data, particularly in the visualization of clustered transcriptomic gene expression patterns. These visualizations take the form of easily generated, yet high‐quality 3D models that can be annotated and rotated in every dimension. These and similar benefits are exemplified by the use of data visualization methods utilized in fields like the physical sciences (Kent 2015), and the recent visualization of single‐cell transcriptomic data within 3D root tissues to demonstrate organ‐specific gene expression (Su et al. 2023). The benefits of 3D projections as opposed to 2D can be seen in Figure 1 when comparing panels A and B, which utilize the same data yet provide very different perspectives. These benefits are further expanded in the HTMLview example dataset S10, which displays *Arabidopsis* transcriptomic data over multiple timepoints. The clear distinction and grouping of genes into specific groups should be noted, especially the gravity that may be lost on 2D projections or through other methods like heatmaps. These methods may be of specific interest for those looking to answer biological questions pertaining to the clustering of transcriptomic data, seen here in the case of the bulk transcriptomic dataset, or differentiation between cell types in the case of single‐cell transcriptomics. Transcriptomic datasets utilizing UMAP have been used directly with interactive techniques such as Virtual Reality (VR), although specialized VR equipment is required for implementation (Legetth et al. 2021). This gap between VR functionality and 2D static images highlights the need for readily accessible 3D visualization technologies for data processed through dimensionality reduction techniques. To address this gap, we have established a method to utilize dimensionality‐reduced data to create both high‐quality and interactive 3D visualizations on computer flatscreens. The method also seeks to provide flexible and ready‐to‐use tools that are not limited to transcriptomic data, in that they can be widely applied.

The methods of visualization presented here utilize a common starting dataset before diverging into two distinct pathways, one based on Blender and the other based on Hyper Text Markup Language (HTML). The Blenderview pathway utilizes Blender, a free, open‐source toolset for generating, animating, and rendering 3D models primarily used in digital art settings, and it has recently been used in a transcriptomic setting to create visualization of single‐cell transcriptomic data (BO Community 2018; Su et al. 2023). Blender provides a wide array of tools and built‐in features; however, we have focused on its modeling, animation, and rendering abilities. This pathway is preferred for datasets that require animation or higher‐quality movies and images based on 3D t‐SNE projections; however, it lacks interactivity once the movies or images are rendered. The major benefit provided by Blenderview is the animation of the t‐SNE's unsupervised clustering decision process as data points are projected into the 3D space and as the visualization proceeds through the algorithm's iterations. Specifically, this feature of Blenderview allows a glimpse into the dimensional reduction algorithm's iterations, visualizing the movement of the 3D points as they are clustered based on similarity, allowing the user to see the” decision‐making” process. This point of” decision‐making” is of specific interest since these dimensionality reduction methods are classified as unsupervised, meaning the user does not play a large role in the placement of datapoints aside from hyperparameter tuning. The HTMLview pathway is geared for datasets that would benefit from increased interactivity and datapoint exploration, rather than movies of the 3D projections, while still using the same 3D t‐SNE data.

In summary, these methods provide a framework for researchers to create quality animations and data visualizations through the application of open‐source digital resources, including Blender v4.1.1 and the script repository JsDelivr. The methods and resources provided were created by wet‐lab scientists and a digital sciences specialist with wet‐lab scientists in mind to bridge the gap for those not adequately versed in coding and Blender. The guide provided here and in the Supporting Information covers the in‐depth creation of both methods, including Blenderview for animation and rendering in Blender and the formatting of datasets for use with the provided HTMLview template.

## 2 Materials and Methods

### 2.1 Transcriptomic Dataset and t‐SNE Generation

The transcriptomic dataset used in this example originated from a plant experiment on the Virgin Galactic Unity 22 mission (Ferl et al. 2023). The significantly differentially expressed genes were identified with the following cut‐offs: Log2(fold‐change) > 1 or < −1 and a *p* value of < 0.05. These Log2(fold‐change) values were then fed into the t‐SNE algorithm via a CSV file with non‐significant values replaced with zero. The t‐SNE was generated using R (4.2.1) with the package Rtsne with default parameters aside from an eta value of 2000 (Krijthe 2021). The clusters were identified using the DBSCAN from the R package fpc with default parameters except for an eps of 2 and MinPts of 10 (Hennig 2024). The 2D t‐SNE was generated utilizing the same data, t‐SNE parameters, and clustering parameters, and the Plotly chart studio web tool was used for visualization (https://plotly.com/).

### 2.2 Blender

The open‐source 3D modeling and animation software Blender is free and available for download from its dedicated website (www.blender.org). The method presented here utilizes version 4.1.1 with no additional assets or plugins.

### 2.3 JsDelivr and HTML Scripts

Jsdelivr (2025) is an open‐source Content Delivery Network (CDN) that hosts files and provides access to scripts and code that originate from other sites, such as GitHub. JsDelivr is used during the HTMLview pathway to call the necessary scripts, three.js and three‐orbital controls.js, which allow for the rendering and orbital controls for the 3D object in HTMLview. The script required for orbital control, three‐orbitalcontrols.js, and for 3D object rendering, three.js, can be found on GitHub (Casati 2019; Herzog 2024).

### 2.4 Computational Resources

The animating and rendering of the Blenderview and HTMLview pathways do not require advanced computational resources and can be conducted with resources available to most researchers. However, more powerful hardware will decrease the chances of crashes and allow faster rendering times. The Blender animation and rendering were completed on a desktop with a Ryzen 7700X CPU, RTX 3070 GPU (8 GB VRAM), and 32 GB RAM.

## 3 Resource Overview

This method was created and tested with an *Arabidopsis* transcriptomic dataset with the t‐SNE dimensionality reduction method (Ferl et al. 2023). In the specific visualization case presented here, the datapoints are the differentially expressed genes identified in a spaceflight experiment in which *Arabidopsis* plants were flown on Virgin Galactic Spaceship 2. The dataset utilized Log2FC values as the basis of the t‐SNE analysis (Ferl et al. 2023), using only those differentially expressed genes with a *p*‐value of > 0.05 and a Log2FC value with an absolute value greater than 1.0 at any portion of the flight. Although the behaviors of differentially expressed genes are visualized here, any data analyzed through the use of UMAP or t‐SNE could use the visualization methods presented herein, including the standard approach of t‐SNE or UMAPs in transcriptomic settings, which utilize expression data directly (Becht et al. 2019; Kobak and Berens 2019).

The production of the input data begins with the analysis of a transcriptomic dataset utilizing a form of dimensionality reduction, like t‐SNE or UMAP. These two methods of dimensionality reduction utilize the same general method of action: reducing a highly dimensional datapoint to 2 or 3 dimensions (Kobak and Berens 2019). The application of these algorithms creates low‐dimensional representations of datapoints whose locations in a 2D or 3D space are informed by both the algorithm's parameters and the dataset's original variables. These algorithms result in projections where datapoints that have similar variable values are generally relatively close to each other in the 2D or 3D space, thereby clustering the data in a lower‐dimensional space based on the behavior of the variables. Once the data has been projected by the algorithm, the coordinates for each point, the 3D locations for each differentially expressed gene in the given space, can be extracted as spatial coordinates that form the basis of the visualization process. These final spatial coordinates are routed into HTMLview for interactive visualization. Spatial coordinates for the entire process or the final iteration are routed to Blenderview for 3D movies and static 3D images. In Blenderview, these initial and successive spatial coordinates are used as locations to generate 3D objects to represent each datapoint, in the case of the present transcriptomics dataset, a single gene. Additional successive coordinates are used to illustrate the datapoint movement over time during the algorithm's iterations. These positions are then added to the Blender animation as keyframes, or anchor points. These keyframes can be thought of as the stopping points that the datapoints reach at the end of each animation. This means that each new iteration or movement of the gene datapoint must be included as a keyframe. These animations become the 3D models that can then be rendered utilizing built‐in rendering engines within Blender. This rendering process is expedited by the use of a Blender template discussed below and provided in the Supporting Information. This workflow allows for the production of a high‐quality 3D animation of either a single static projection of the dataset at any point of the algorithm or an animated movie of the dimensionality reduction iterative process as the algorithm processes the data.

In HTMLview, only the final iteration of 3D spatial coordinates of a t‐SNE or UMAP, which are most often seen in transcriptomic‐related publications, is used for the visualization. These spatial coordinates are similarly processed into an accessible format for the HTMLview templates provided in Supporting Information. This HTMLview pathway produces an interactive HTML file that the user can manipulate in real time to examine different portions of the 3D environment. The pathway also allows for simple incorporation of metadata, such as hover information for points, and other labeling and hyperlinking. After the HTML template has been completed, the model is rendered in an interactive 3D space, which is enabled through the use of scripts hosted on the open‐source code sharing network JsDelivr (see Supporting Information).

**FIGURE 2:**
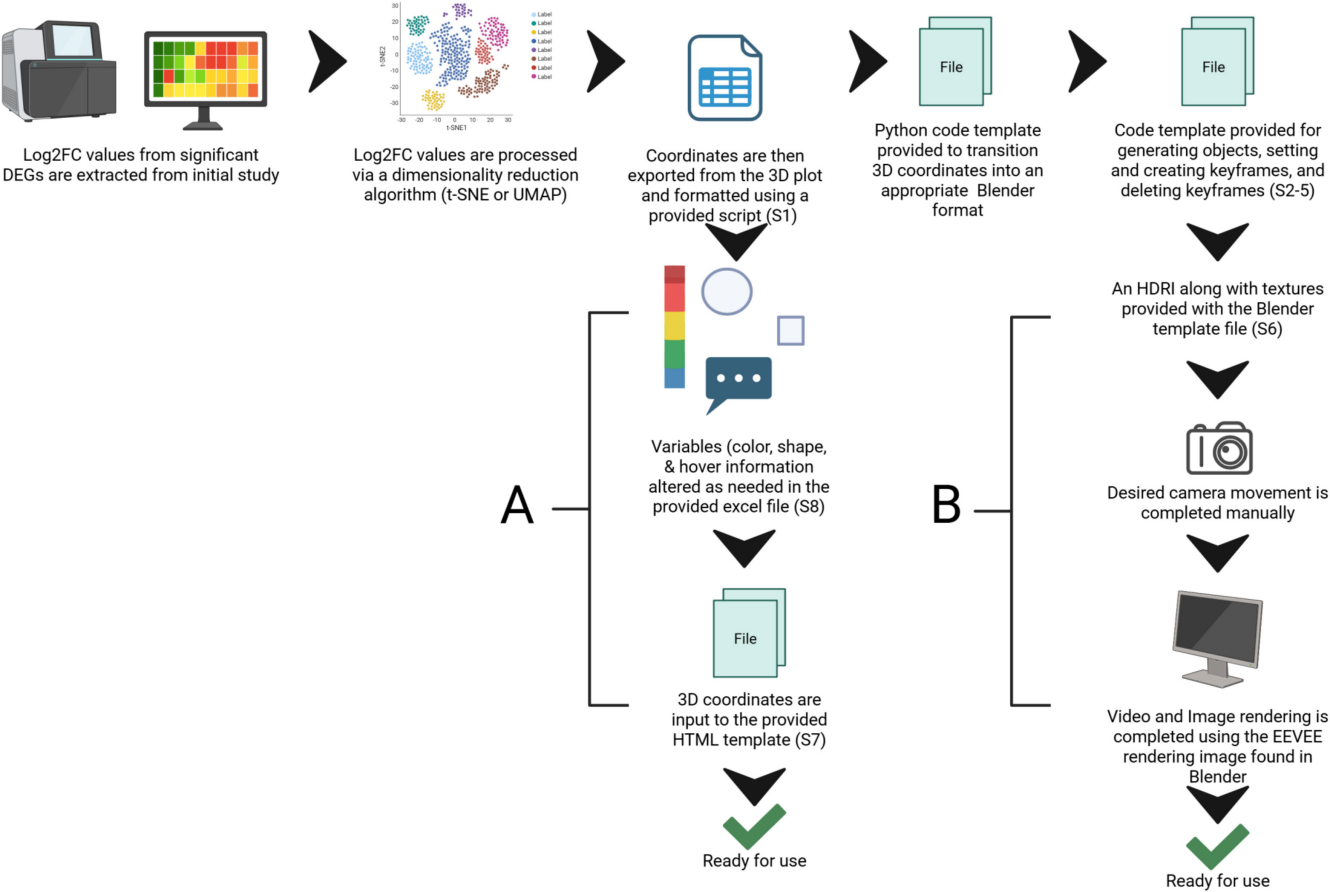
Workflow illustrating the two provided pipelines. Both pipelines are fed from typical transcriptomic data that are reduced to 3D plot coordinates. The Blender pipeline to the right of the diagram is simplified into its most important components including coordinate conversion, object generation, keyframe setting, HDRI deployment, camera movement, and rendering. The HTML pipeline in the middle of the diagram is a much more basic and less resource heavy workflow consisting of coordinate input and variable refinement. The workflow can also be broken down into two levels of difficulty, beginner and intermediate, which are denoted by (A) and (B), respectively. This figure was created in https://BioRender.com.

The scripts S1–S6, templates S7–S8, guide S9, and HTMLview example S10 on how to apply the method presented here are included in the Supporting Information along with public access on GitHub (made public upon publishing), along with the general workflow illustrated in Figure 2. The scripts are provided in the order in which they should be used. The first S1 is used to convert the 3D coordinates obtained from a t‐SNE or UMAP analysis into coordinates that are appropriately formatted for use with the Blenderview and HTMLview pathways outside of Blender, in Python. Four of the files provided in the Supporting Information are for exclusive use with Blenderview S2–S6. To generate the 3D shapes to represent a datapoint, in this case a unique gene, a script is provided S2 to use in Blender's scripting window. The first keyframe must be set using the coordinates that were used to generate 3D objects in the Blender space, S3. If an animation is desired, additional keyframes with coordinates must be input into Blender with the script provided, S4. In case we utilized t‐SNE iterations to highlight the algorithm's process. We also provide the necessary script to eliminate unwanted keyframes or those generated by mistake, S5. It is important to note that deleting keyframes is more difficult than creating them and may cause Blender to crash if many points are being affected. These actions can be completed in a new Blender file or run in the provided Blender file template S6, which also has a custom gridded High Dynamic Range Image (HDRI) background. The remaining supplementary files S7 and S8 are used exclusively for the HTMLview pathway. We provide an HTML file template S7, which includes all the necessary code required to generate the HTML after the coordinates have been properly formatted and data have been annotated using the Excel file provided S8. We also provide an existing *Arabidopsis* bulk transcriptomic dataset from Ferl et al. (2023) formatted using the HTMLview method as a point of reference S10.

## 4 Results and Minor Discussion

The difference between the two projections, 2D and 3D, seen in Figure 1 illustrates the utility of the method provided herein through the increase in data interactivity and model visualization quality. The process was refined using an *Arabidopsis* transcriptomic dataset with the t‐SNE method of dimensionality reduction, with the workflow outlined in Figure 2 (Ferl et al. 2023). The flexibility of the method allows for the application of virtually any dataset utilizing methods of dimensionality reduction, like t‐SNE or UMAP, which can generate 3D coordinates.

**FIGURE 3:**
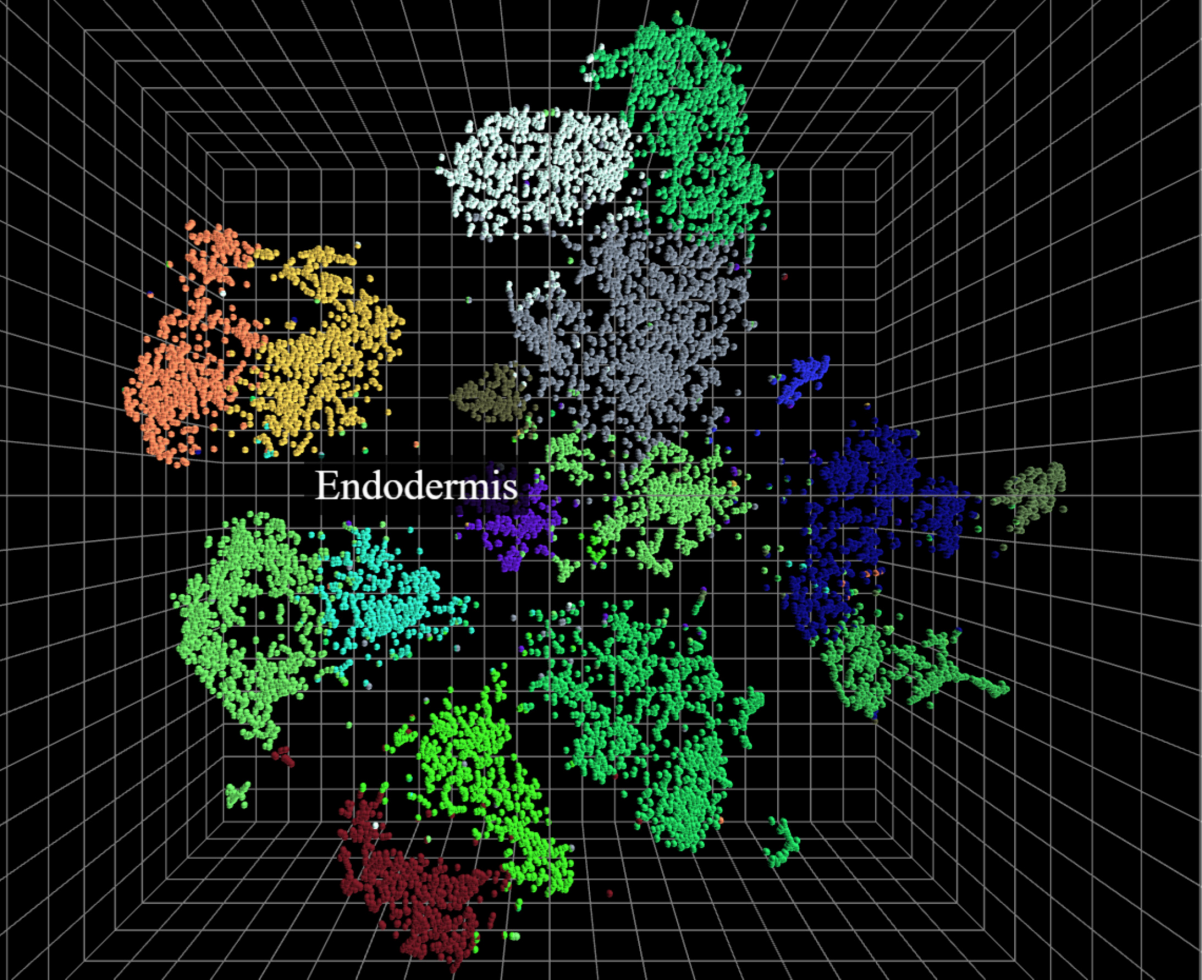
HTMLview proof of concept with *Arabidopsis* root single‐cell transcriptomic data. *Arabidopsis* root single‐cell transcriptomic data, and t‐SNE metadata, was utilized from Shulse et al. (2019). It was processed using the HTMLview method. The 17 clusters identified by the original analysis were then colored with an arbitrarily picked color scheme. The metadata component of the HTMLview pipeline also added the ability to place cell type identification information into the hover‐over information for each cell.

Implementation of these methods for single‐cell datasets is relatively simple and relies solely on the extraction of 3D coordinates from the t‐SNE or UMAP object. A brief example of how to extract these datapoints is provided in the overall guide, S9, as the first step in both the HTMLview and Blenderview methods. The primary goal is simply the extraction of the datapoints 3D coordinates (X, Y, Z) following the dimensionality reduction algorithm's execution. These coordinates are then taken straight to either supplementary file 1, S1 for reformatting of the points into acceptable input formats for either method, although this reformatting could also be completed manually. As proof of concept, we have utilized the single‐cell root transcriptome dataset from Shulse et al. (2019) to create an HTMLview visualization in Figure 3. The dataset consisted of 12,198 *Arabidopsis* root cells, which were processed using t‐SNE, with 17 clusters then identified (Shulse et al. 2019). This example took approximately 35–40 min to complete, exemplifying the speed and utility of the HTMLview method. Note that to project the 2D data into the 3D space, a Z axis value of zero was added for all of the datapoints.

While the two methods, Blenderview and HTMLview, are relatively flexible, there are computational bottlenecks to be aware of. Regarding Blenderview, the total number of objects will cause script execution times to increase. For example, animating the movement for > 10,000 points can result in code execution times in the range of 10–20 min per script. This fact is also true when generating large numbers of points > 5000 at a time, meaning that it would be best to generate or edit them in batches or groups when working with large datasets. It may also be of note that in the cases of these large datasets > 50,000 points, it may be best to group these points in Blender so they are treated as a single object to reduce computational strain, which would only pose issues if animations for specific points are desired. However, this limitation is largely dependent on your machine's hardware specifications and can be largely ameliorated through access to better graphics cards and memory resources. In some cases, universities have hypercomputing resources available with Blender already available on their network. HTMLview has similar limitations with increasing numbers of points, resulting in lag while moving within the 3D space. This is more readily remediated with performance optimizations than in Blender, which primarily consists of reducing segments or vertices on objects like spheres and text. This reduction in vertices can result in objects not looking smooth, but only when taken to the extreme. For instance, the vertex parameter can be reduced from 32 to 16 to virtually no ill effect, the vertex parameter being the last two numbers within the “sphereGeometry” constant. There are other more advanced methods of removing these bottlenecks in the HTMLview pathway, such as causing points that are not in view to not be projected within the space, which greatly reduces the strain on computational resources. There may also be more aesthetics‐based issues, such as point occlusion; these will likely need to be addressed on a point‐by‐point basis through modifications such as point opacity or arbitrary modifications to 3D coordinates, like increasing them all by a factor of 3 to maintain relationships but create more open space between points.

**FIGURE 4:**
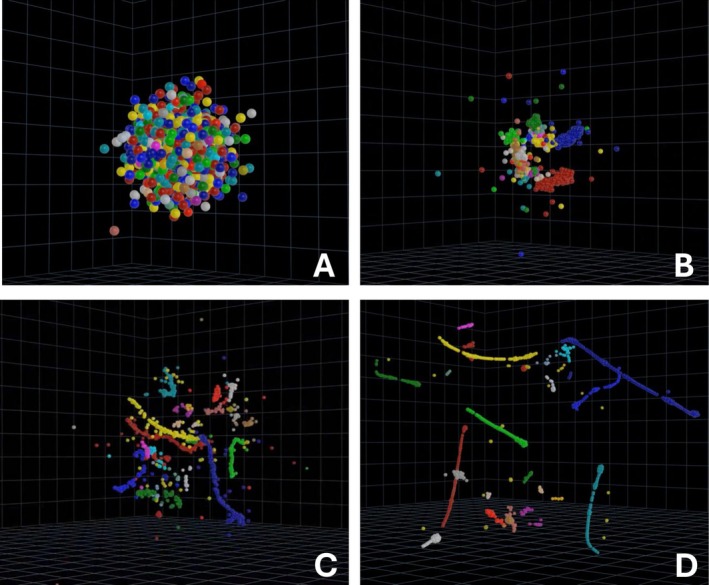
The final rendered product of the Blender workflow. The fully rendered Blenderview shows the points movement in 3D space as the t‐SNE proceeds through increasing iterations until a final projection is determined (A–D). Here the points from the 3D t‐SNE are exported, spheres are generated in the rendering space, and then they are moved to specific 3D coordinates provided by the different iterations which are set as keyframes. The full video associated with these images is provided in the supplementary section, S11.

**FIGURE 5:**
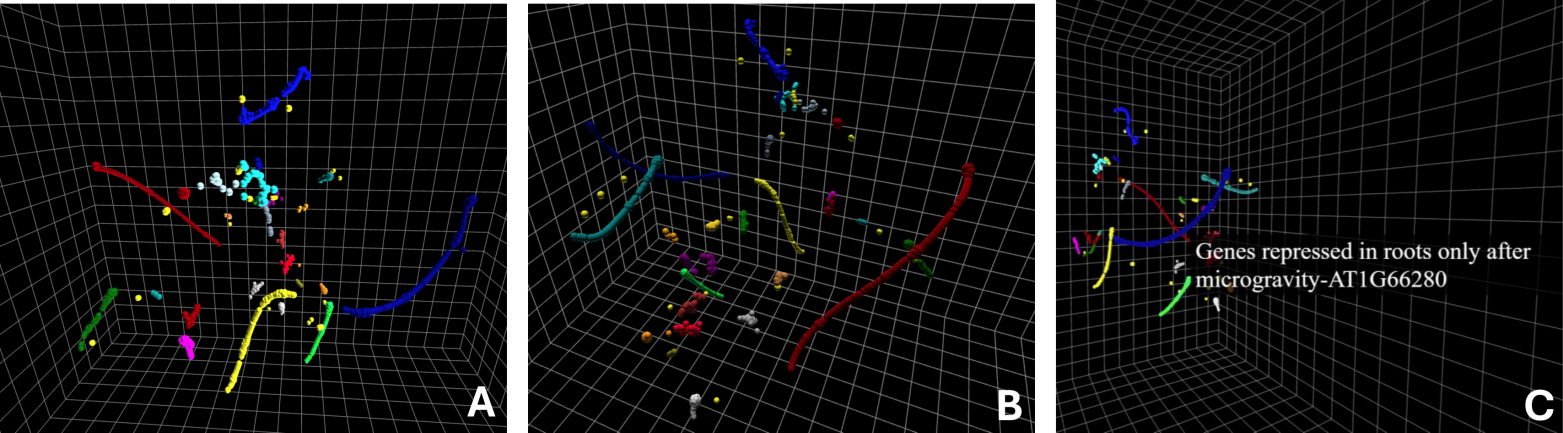
The final product of the HTML workflow using the provided template. The images were taken from various angles using the html file's click and drag abilities (A,B). Along with the implementation of additional capabilities of hover information (C), in order to display in real time additional data from any point in the display. An interactive, downloadable version can be found in the supplemental file 10, S10.

The Blenderview pathway allows for the production of animations both to illustrate the t‐SNE algorithm's iterative process and for a more in‐depth analysis of clusters enabled through camera movement. In Figure 4, the Blenderview pathway was utilized to visualize all 1401 differentially expressed genes from the original *Arabidopsis* transcriptomic dataset. The animation of keyframes from several iterations allows for a window into the algorithm's decision‐making process, illustrating some of the dataset's key components. The HTMLview method of utilizing a pre‐prepared HTML file can be seen in Figures 3, 5, and supplementary file 10, S10. HTMLview provides a more interactive and easily accessible look into a dataset due to the audience's ability to freely investigate the datapoints and the relative ease to generate. The benefits and limitations of both methods can be seen comparatively in Table 1. It is worth noting that both of these methods stand apart from previously proposed methods that project a 3D model of a dataset using VR for visualization due to their ease of use and lack of required specialized equipment. Overall, the method provided has allowed for the production of both high‐quality animation and videos utilizing Blender and interactive HTML files, allowing an audience to dive deeper into transcriptomic datasets more easily than before. The method allows those with and without coding or animation experience a straightforward entry point into generating and animating 3D models in Blender or a ready‐to‐use HTML file for use with a wide array of datasets.

**TABLE 1:** Method Comparison between Blenderview and HTMLview.

Method comparison		
	Blender view	HTML view
Features	High quality animations, Higher quality aesthetics, Greater range of aesthetic choices (textures, lighting, etc.)	Interactivity, Explorability, Easily manipulate existing datapoints
Limitations	Requires additional software and potentially computing resources, Increased difficulty for editing datapoints, Rendering time required	Lacks animations, lower quality of aesthetics
Benefits	Higher quality aesthetics, Outputs easily incorporated into webpages or presentations	Ready‐to‐use, Easy file sharing, No rendering time required

*Note:* The table provides a brief summary of the primary features, limitations, and benefits. These distinctions are provided to better enable potential users to identify which method will best fit their purposes.

## 5 Conclusions

Data visualizations are a key component of scientific communication and play a role in all areas of biological research. The need for attractive and interactive data visualization methods has only seen growth as the prevalence of high‐throughput data requiring dimensionality reduction methods, like single‐cell RNA sequencing, has increased. Here, we have introduced the use of non‐standard tools, like Blender, to enable users to generate interactive and engaging models to communicate data. With regard to answering specific biological questions, these methods provide help project data in ways that are more palatable to the human eye, allowing researchers to better visualize and communicate significant features among 3D projected data, whether it be clustering based on Log2FC as seen in the example dataset or based on read counts in single‐cell RNAseq like that in Figure 5. The method could also be easily retrofitted for other uses under the condition of being able to create or access 2D or 3D coordinates. These potential uses include the projection of additional graphs that are not focused on dimensionality reduction, like volcano plots and other coordinate‐based plots. This flexibility allows for applications that extend beyond exclusively transcriptomic‐focused approaches. The two pathways provided also cater to different levels of difficulty and effort, with the HTMLview path being quick and relatively easy and the Blenderview path requiring additional effort. Much of this effort has been reduced via the supplemental guide and the provided Blender template file, which simply needs to be populated with data points and rendered to be complete. Neither of the two pathways requires extensive computational resources, with the HTMLview path being able to be completed on machines capable of using a simple text editor and a web browser, and the Blenderview pathway largely relying on a graphics card, which is commonly available in academic or research settings. The implementations of the end products are quite flexible, with both the HTMLview and Blenderview models being readily deployable through the use of linked QR codes or embedding into webpages. This further expands on the utility of the method by providing the avenue for audiences to either be visually guided through a dataset in real time through a Blenderview animation or explore an interactive dataset firsthand, in the case of the HTMLview file.

## Author Contributions

H.F.S. created the HTML pipeline and contributed to the Blender pipeline. A.S. created the majority of the Blender pipeline. R.F. and A.L.P. designed the concept and supervised the project. H.F.S. and R.F. wrote the manuscript with input from all authors. All authors reviewed, edited, and approved the manuscript.

## Supporting information


**Appendix S1:** Supporting Information.

## Data Availability

All external resources used have been linked with the methods section. Scripts used for both the Blenderview and HTMLview methods have been provided in the supplementary section and within a GitHub repository that will be made publicly available upon the acceptance of this manuscript (https://github.com/UFSpacePlants/Digital‐resources‐for‐3D‐visualization‐of‐clustered‐transcriptomic‐data).

## References

[ppl70500-bib-0001] Becht, E. , L. McInnes , J. Healy , et al. 2019. “Dimensionality Reduction for Visualizing Single‐Cell Data Using UMAP.” Nature Biotechnology 37: 38–44. 10.1038/nbt.4314. 30531897

[ppl70500-bib-0002] BO Community . 2018. Blender—A 3D Modelling and Rendering Package. Stichting Blender Foundation.

[ppl70500-bib-0003] Casati, G. 2019. Three‐Orbital Controls.Js, Github Repository. https://github.com/fibo/three‐orbitcontrols/.

[ppl70500-bib-0004] Ferl, R. J. , M. Zhou , H. F. Strickland , et al. 2023. “Transcriptomic Dynamics in the Transition From Ground to Space Are Revealed by Virgin Galactic Human‐Tended Suborbital Spaceflight.” Npj Microgravity 9: 1–11. 10.1038/s41526-023-00340-w. 38123588 PMC10733374

[ppl70500-bib-0005] Hennig, C. 2024. FPC: Flexible Procedures for Clustering. R Package Version 2.

[ppl70500-bib-0006] Herzog, M. 2024. ‘Mrdoob/three.js’. https://github.com/mrdoob/three.js.

[ppl70500-bib-0007] jsDelivr . 2025. A Free, Fast, and Reliable CDN for JS and Open Source jsDelivr. https://www.jsdelivr.com/.

[ppl70500-bib-0008] Kent, B. R. 2015. 3D Scientific Visualization With Blender. Morgan & Claypool Publishers.

[ppl70500-bib-0009] Kobak, D. , and P. Berens . 2019. “The Art of Using t‐SNE for Single‐Cell Transcriptomics.” Nature Communications 10: 5416. 10.1038/s41467-019-13056-x. PMC6882829 31780648

[ppl70500-bib-0010] Krijthe, J. R. 2021. Wrapper for Van Der Maaten's Barnes‐Hut implementation of t‐Distributed Stochastic Neighbor Embedding. R package version 0.16.

[ppl70500-bib-0011] Legetth, O. , J. Rodhe , S. Lang , P. Dhapola , M. Wallergård , and S. Soneji . 2021. “CellexalVR: A Virtual Reality Platform to Visualize and Analyze Single‐Cell Omics Data.” iScience 24: 103251. 10.1016/j.isci.2021.103251. 34849461 PMC8609247

[ppl70500-bib-0012] Shulse, C. N. , B. J. Cole , D. Ciobanu , et al. 2019. “High‐Throughput Single‐Cell Transcriptome Profiling of Plant Cell Types.” Cell Reports 27: 2241–2247. 10.1016/j.celrep.2019.04.054. 31091459 PMC6758921

[ppl70500-bib-0013] Su, C. , M. Lyu , A. P. Mähönen , Y. Helariutta , B. De Rybel , and S. Muranen . 2023. “Cella: 3D Data Visualization for Plant Single‐Cell Transcriptomics in Blender.” Physiologia Plantarum 175, no. 6: e14068. 38148248 10.1111/ppl.14068

[ppl70500-bib-0014] van der Maaten, L. , and G. Hinton . 2008. “Visualizing Data Using t‐SNE.” Journal of Machine Learning Research 9, no. 11: 2579–2605.

