# Supplementary material for: Application of Open‐Source Digital Resources for 3D Visualization of Clustered Transcriptomic Data
Source: Physiol Plant. 2025 Sep 17;177(5):e70500. doi: 10.1111/ppl.70500 (PMC12441758; doi:10.1111/ppl.70500)
Supplement: Supplementary file 1 — Appendix S1: Supporting Information. [file PPL-177-e70500-s001.zip › ppl70500-sup-0001-Supinfo/S_10 HTMLview Example Dataset.html]

Virgin Galactic Bulk Plant Transcriptome Example
