# Supplementary material for: Application of Open‐Source Digital Resources for 3D Visualization of Clustered Transcriptomic Data
Source: Physiol Plant. 2025 Sep 17;177(5):e70500. doi: 10.1111/ppl.70500 (PMC12441758; doi:10.1111/ppl.70500)

**Blender Resource Guide (Supplemental)**

1. The first step for both methods, Blender video rendering and interactive HTML, is to extract the 3D coordinates from the t-SNE, or UMAP, output. In our case the t-SNE was generated using the Rtsne package in R with the file. To save the coordinates to a usable CSV file the example code below was executed in R after the t-SNE algorithm has finished.


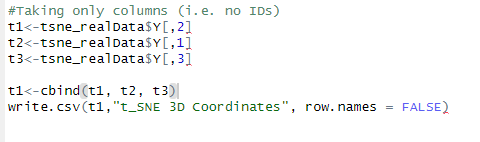


The image above saves the X, Y, and Z coordinates as data objects which are then concatenated together with “cbind” before being written to a usable output with “write.csv.”

1. The second step is to process the coordinates into usable 3D coordinate trios which can be used in both Blender and the HTML files. This task is rather tedious and can be completed by hand using Microsoft Excel or with additional code to help automate the process. The code below (S_1 Code for Converting 3d coordinates to a list (Do not run in Blender).txt) utilizes the CSV output to put the coordinates into the correct format for the downstream processes.


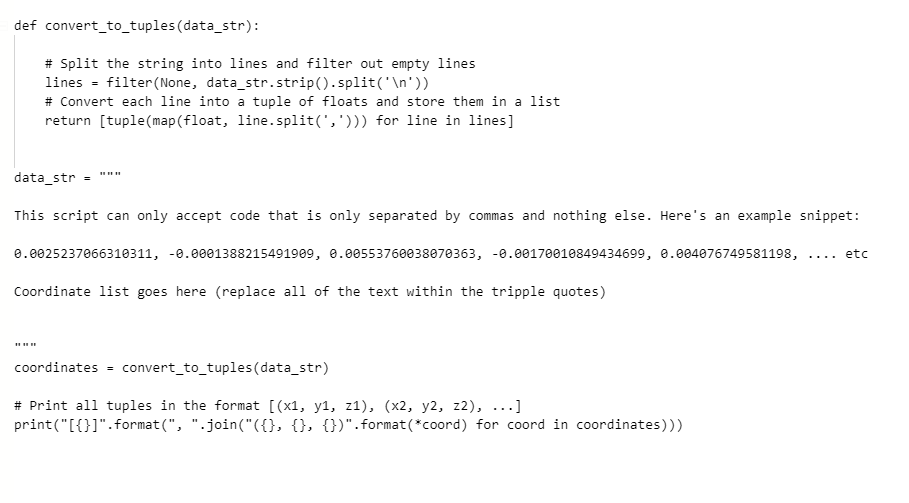


1. These coordinates can then be fed directly into the Blender datapoint visualization script or amended with additional metadata such as color. The example below illustrates the script ( S_2 Code for Spawning Intial Spheres.txt) for generation of 5 points with varying colors within blender. This can be used to generate a 3D model of the t-SNE output or as the beginning frame to illustrate the t-SNE’s iterative process. Keep in mind that you must put the correct color at the correct point in the list for the sphere to be colored correctly. For example, if the sphere whose coordinates are listed third in the list the color for that sphere must be third on the “colors” list.


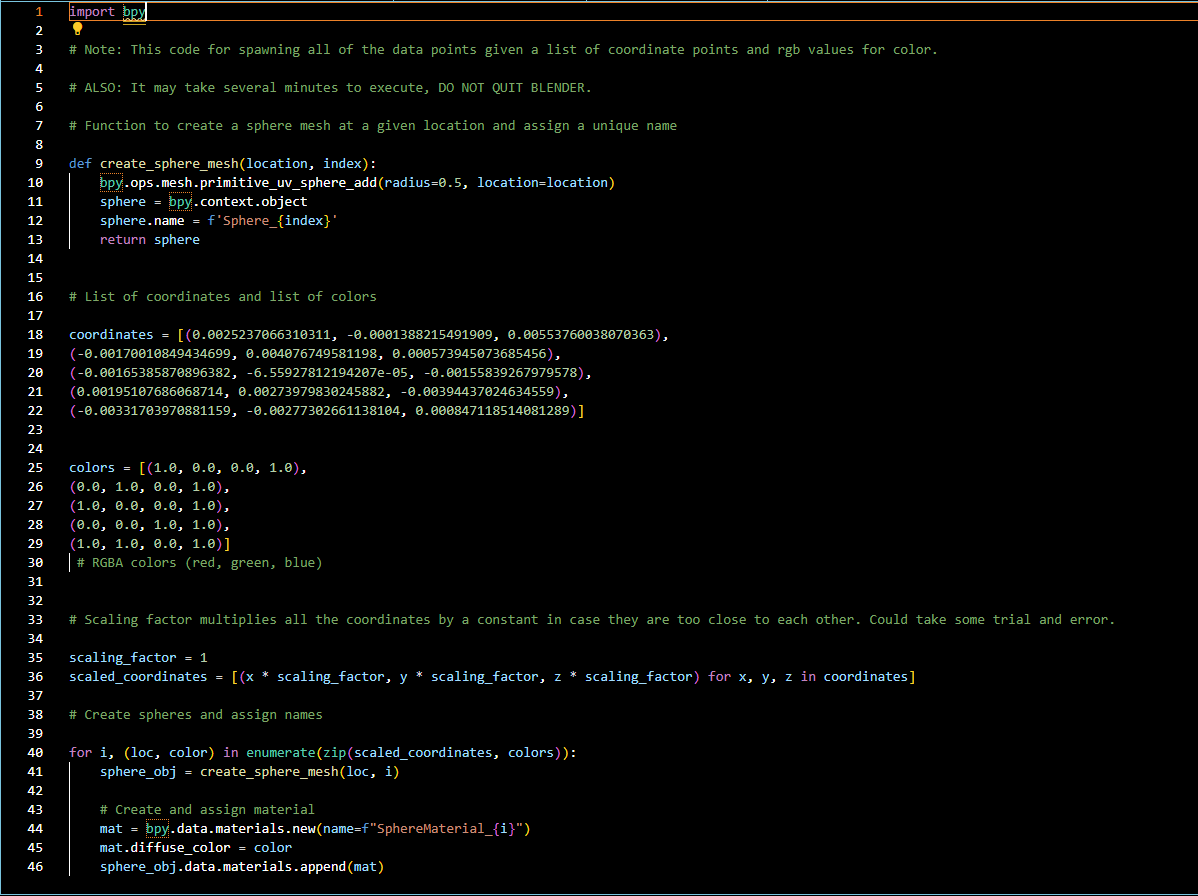


This code will then be copy-pasted into the scripting tab found along the top ribbon in Blender after opening the tab and selecting “new”, seen in red below.


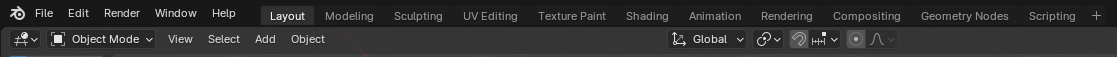

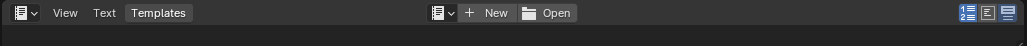


1. To continue further and illustrate the t-SNE’s iterative process you will need to re-run the t-SNE algorithm using a predetermined number of iterations. In our case, we re-ran the t-SNE algorithm and set the “Max_iter” parameter in intervals of 20 until reaching 560, an approximate copy to the final iteration. You may need to employ the “set.seed()” parameter during the production of these iteration coordinates to maintain consistency among the iterative projections. These coordinates will then be processed in the same fashion as the previous coordinates but will be entered into blender via a different script. When utilizing coordinates from very early iterations it may be necessary to alter the scaling factor of the spheres so that you may still see unique spheres and not a large mass of spheres.
2. After the initial spheres have been generated their current position must be set as the first keyframe, or animation anchor point, with the code below (S_3 Code for setting first keyframe.txt). This code will set all of the existing sphere’s current coordinates as the first keyframe.


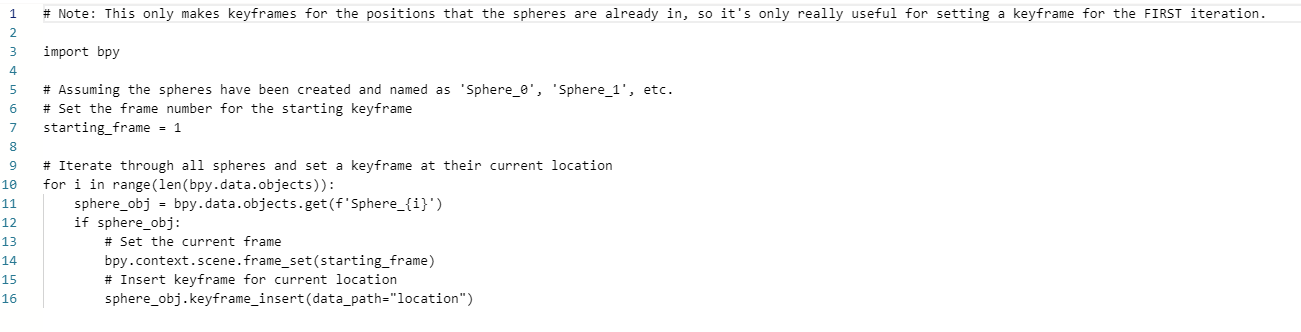


1. To create an iterative animation, you must then create the keyframes for the following coordinates the spheres should move too created from the exported coordinates discussed earlier. The code below (S_ 4 Code for setting any keyframe.txt) can be used to generate new keyframes. Each keyframe will represent one iteration point, so each iteration you would like to include should have its own dedicated keyframe.


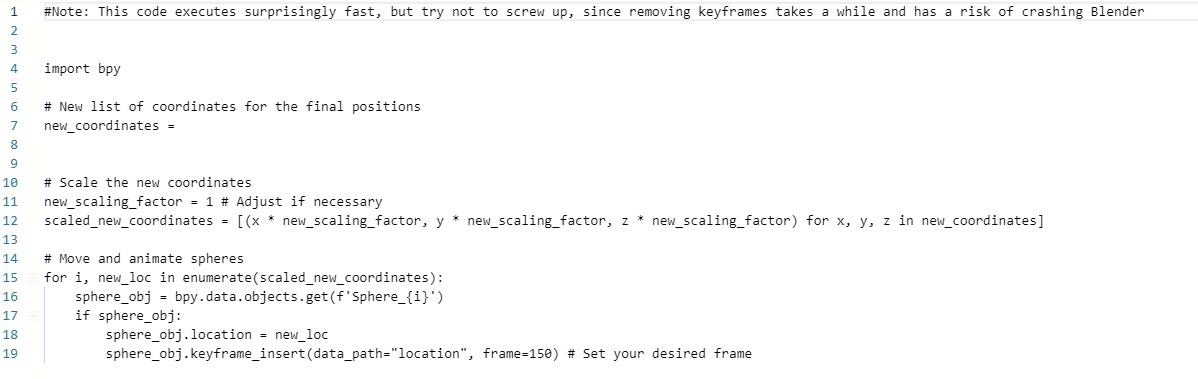


1. Keyframes can be removed using the code below (S_5 Code for deleting keyframes.txt). This code may cause issues with Blender and has been known to cause the software to crash completely.


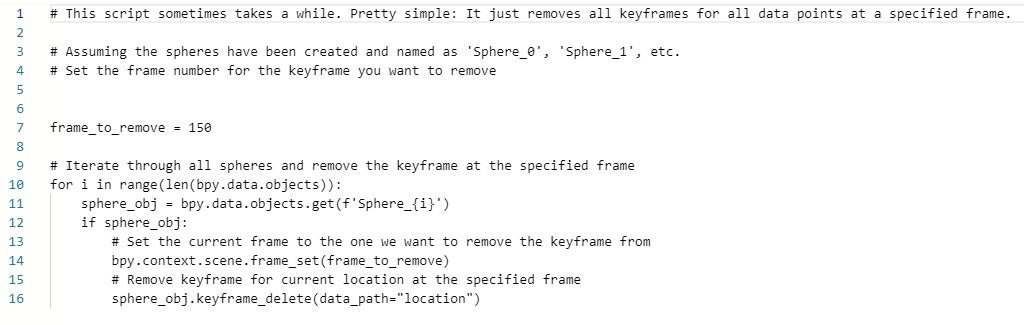


1. In order to have a video output of the animated data, a camera with several modifiers needs to be in the 3d scene. In Blender, cameras function very similarly to their real-world counterparts, and as such must have empty objects to serve as focal points. In addition, an empty object to serve as a directional guide for the focal point is typically required for easier animation. The empty object can be added to the scene by clicking add in the second ribbon at the top of the screen and then choosing an empty object of your choice, like sphere or cube seen below. The focal point, empty object, can then be attached in the camera settings tab, under the “Depth of Field” section, which can be found by clicking the green camera icon on the bottom right of the screen.


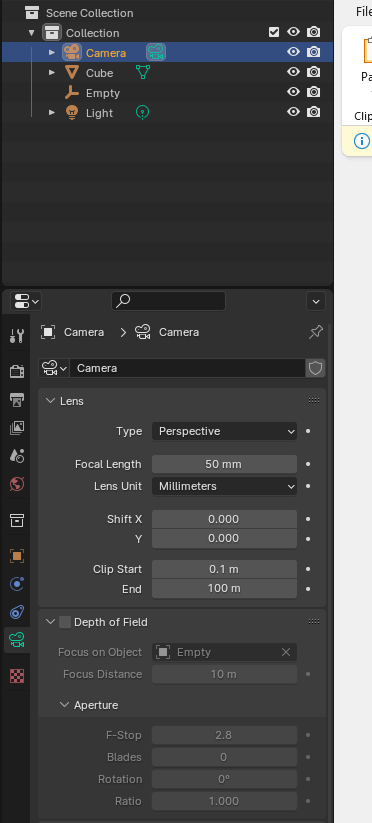

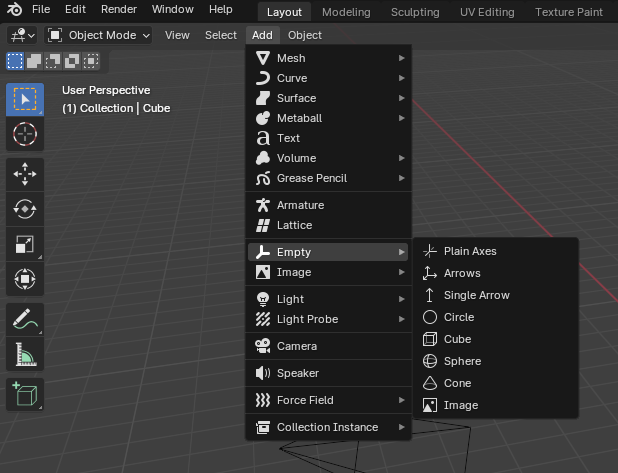


1. The directional guide, the object camera will be facing, is attached by adding a “Track To” constraint in the constraints tab, and then selecting the directional guide object as a reference.


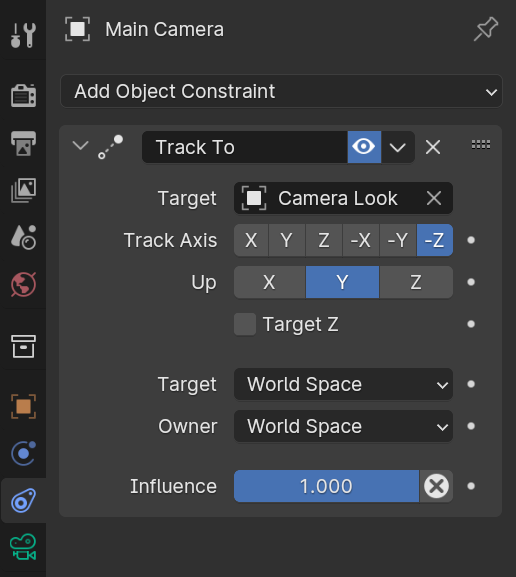


1. The camera, focal point, and directional guides can all have their positions keyframed and animated via the graph editor to create smooth animations focused on specific data clusters. To add a keyframe, simply right click an any axis (x, y, or z) in the “transform” tab and click on “insert keyframe”. These keyframes can then be seen in the graph editor, which shows how blender’s animation algorithm interprets the movement between the keyframes on each axis.


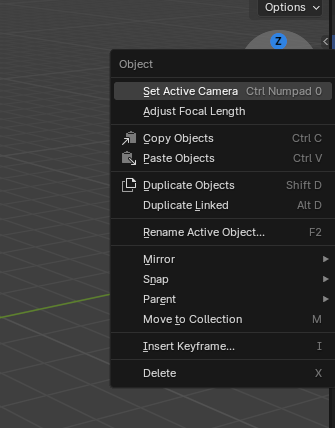


1. To access the graph editor tab, click the clock image at the bottom of the layout tab screen above the frame playback ribbon. Then click on graph editor from the animation column.


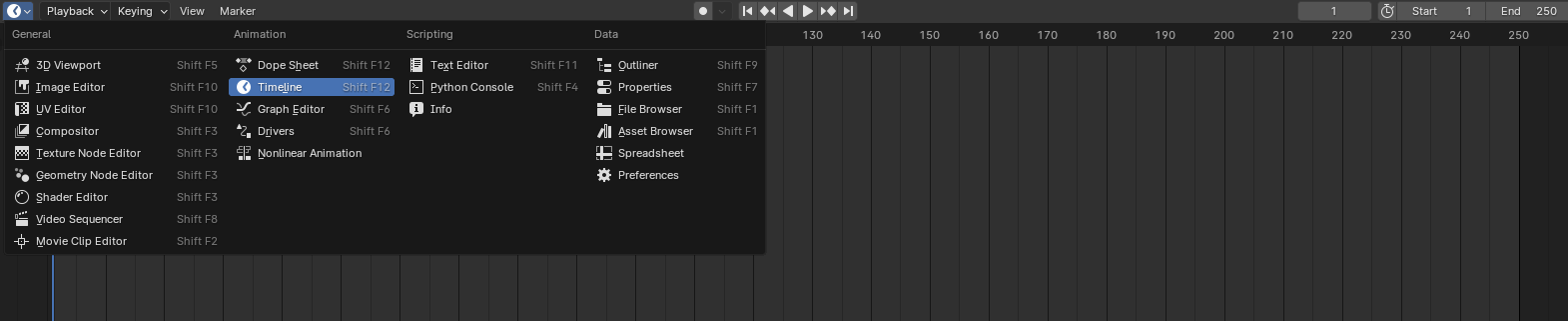

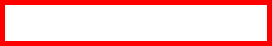


1. The addition of keyframes will add curves that have two open circles near the keyframes, called “handles”, and can control the curves between keyframes. This can either smooth or sharpen the transition between them. The actual keyframes themselves can also be moved by selecting them. It is recommended to lock the selected keyframes on a specific axis when they are being moved, which can be done by pressing “G” (shortcut for grab), then pressing “X” or “Y” (to lock its movement on the x or y axis). The process of adding and adjusting keyframes is similar to animating the camera, focal point, and directional guide. Adding and adjusting keyframes for each as needed will eventually yield the desired animation, which can be seen in action via the playback bar.
2. The simplest and most effective way to get proper lighting in a 3D environment is through using a High Dynamic Range Image (HDRI), as a reference. HDRI images are high-resolution pictures that are designed to wrap around a sphere and are primarily used as backgrounds for 3D scenes. There are numerous websites online that offer a wide variety of free high quality HDRI images. For this application, lighting that is relatively even and neutral in color is recommended. To apply this lighting to the scene, navigate to the “Shading” tab, and change it from “Object” shading to “World” shading, seen in red below.


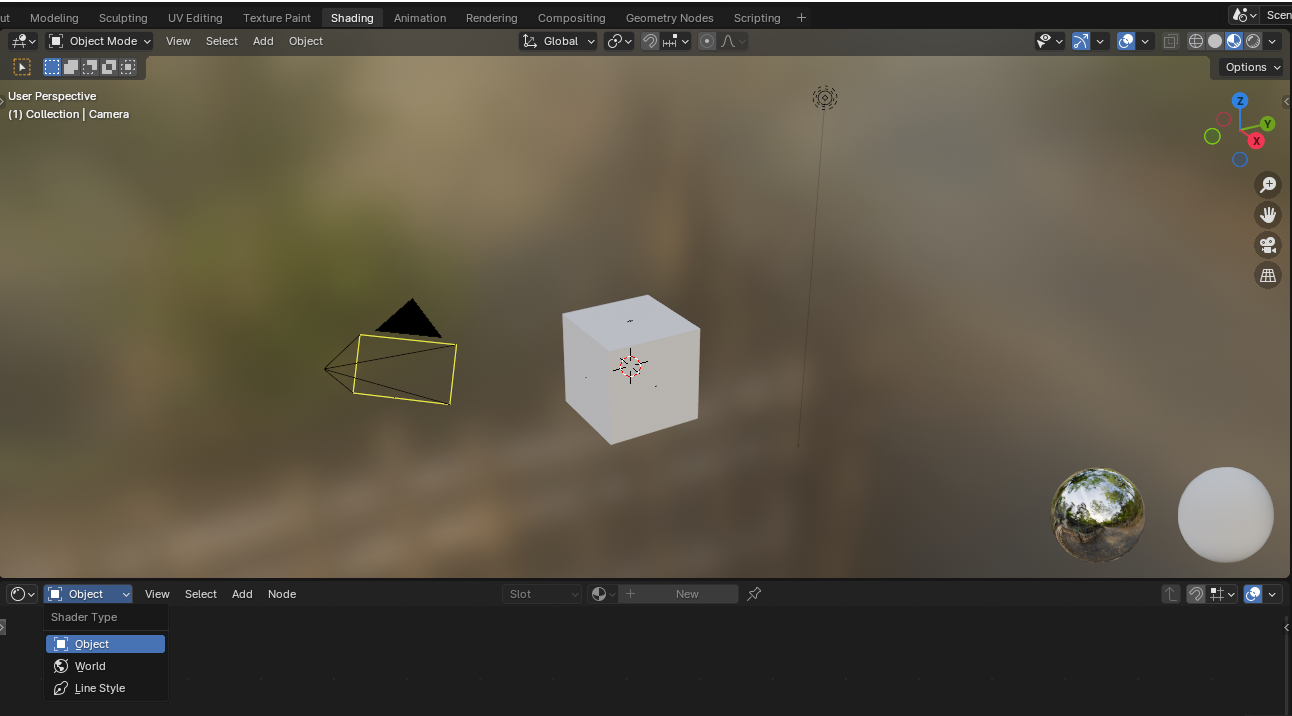


1. Once there, copy the node tree below as closely as possible (pressing “shift” and “a” will allow for searching for specific nodes and will make this process very simple). Though appearing complex, there are only a few key nodes of interest for controlling the scene lighting. The most important one is the large orange "background texture” node in the center, which controls which HDRI image is used for the lighting. The green “background” node that the image node connects to is what controls the strength of the lighting, with a higher number corresponding to brighter lighting. Finally, the blue “mapping” mode controls the orientation of the lighting image and can be modified if needed (though usually this is not necessary). If any background other than a solid color is required, the bottom “background” node can be replaced with another “background texture” node.


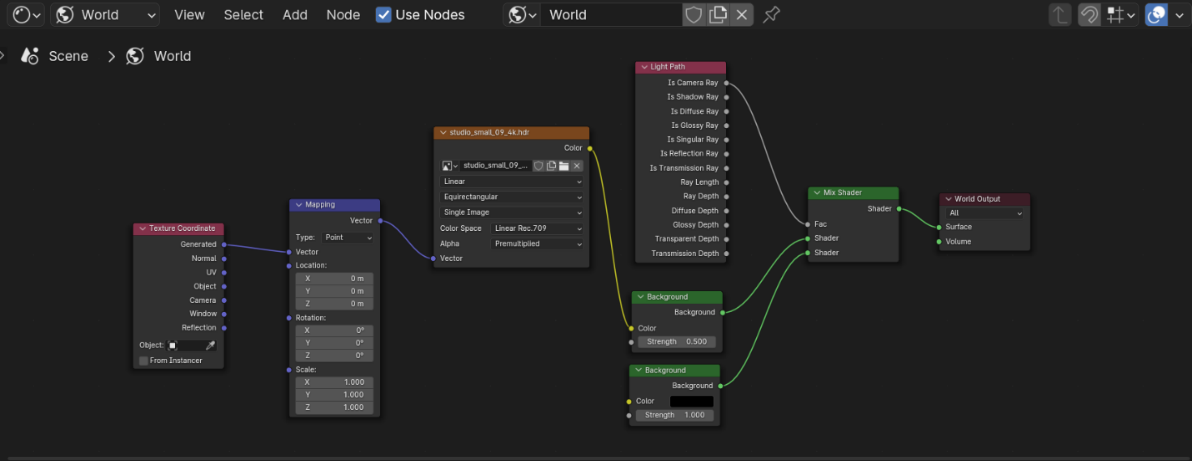


1. Text: To add text to a Blender scene, navigate to the top of the UI and select “Add” and then “Text”. A default text object should spawn in at coordinates (0,0,0). Each text object functions similarly to a text box found in software like Word or PowerPoint. To edit the contents of the text object, simply press “Tab” to toggle edit mode and type as needed.


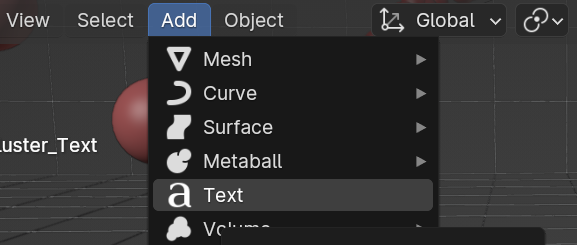


1. In order to change the size, depth, or font of the text, navigate over to the text tab (as seen below). The text can be changed to any font that is stored locally on the computer, and the depth of the text can be edited in the “geometry” section under the “extrude” bar. Changing the size of the text is just a matter of selecting the text object and pressing “S” (shortcut for “Scale”, and is useful for quickly increasing/decreasing the size of objects).


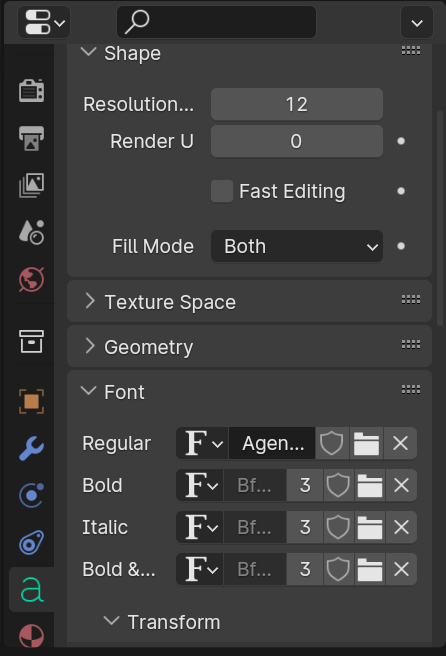


1. To edit the text texture, navigate to the shading tab and replicate the node tree (as seen below). This node tree allows for the text to be any solid color, which can be edited in the “Principle BSDF” node under “Base Color”, and “Strength” (under the Emission section) controls how much the text glows. The second Color bar at the bottom controls what color the glow effect is (which can be different from the color of the object if needed). Other than that, the only other area of interest is the “Mix Shader” node, which controls how visible the text is in the scene and allows it to disappear and reappear when needed. When the “Fac” bar is at 0, the text is fully visible, and when the bar is at 1, the text is fully invisible. This can be keyframed by selecting the “Mix Shader” node, right clicking on the bar, and selecting “Insert Keyframe”. It’s important to note that these keyframes will only show up in the graph editor if the mix shader node itself remains selected.


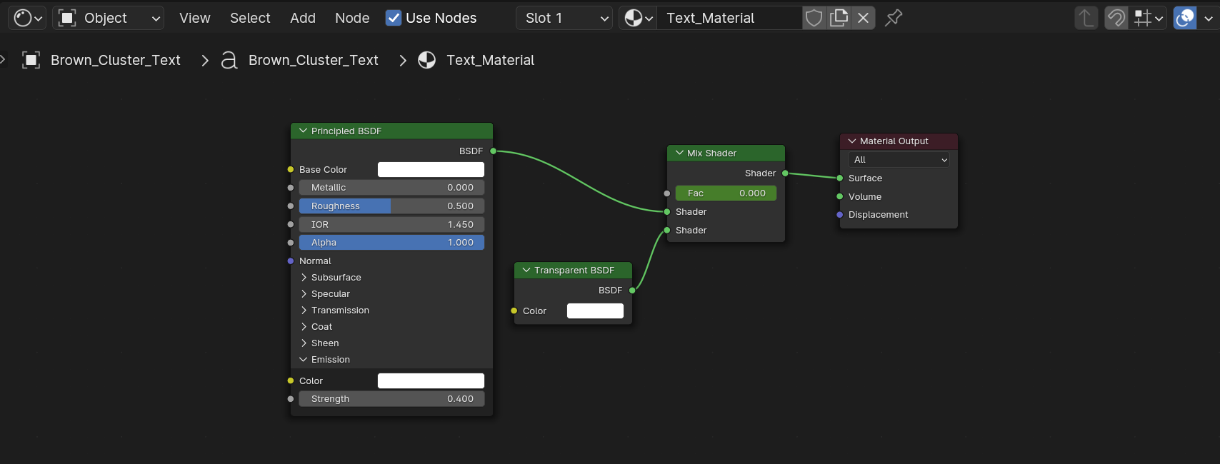


Finally, it’s important to note that the text itself will only properly fade in or out of visibility if the material blend mode is in “Alpha Hashed” or “Alpha Blend”. The default mode of “Opaque” will abruptly fade in or out and will leave behind a dark shadow when the text is supposed to be fully invisible.


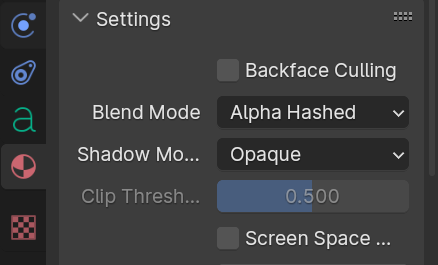


1. Once the animations and lighting settings are all completed, the scene can be rendered into an mp4 video. Blender has two rendering engines: EEVEE and Cycles, which can be toggled in the render settings tab. While both have their strengths and weaknesses, EEVEE would be the recommended choice in this application for its faster speed and efficiency (for reference, the same animation that takes EEVEE 40 minutes to render can take up to 3 hours if rendered using the Cycles engine). Aside from the rendering engine, most of the other render settings can be left as default (unless specific visual effects like bloom and motion blur are needed). The time required for rendering depends on the computer hardware available, with the most important factor being the GPU (graphics card). The more powerful the GPU, the faster the animation will render. The final step is to adjust the output settings, which are found in the “output” tab right below the render tab. In this tab, the resolution, frame rate, and file format/location can be adjusted. The choice for these options ultimately comes down to the specified project requirements, typically video outputs should be in a 1920p by 1080p or a 3840p by 2160p resolution, and the framerate should be either 24 or 30 frames per second. The recommended file output type is an “FFmeg Video” (which is another way to say mp4 video). All these settings should be verified to be correct before rendering starts. To start the render, navigate to the top of the window and click on “render”, then “render animation”. Once the render begins, a second window will appear to show each frame being rendered. It is recommended that the computer is not used for anything else during this time, since it will be dedicating all its available resources to rendering the animation, and any signification interruption could cause the computer to crash and the render to be aborted.


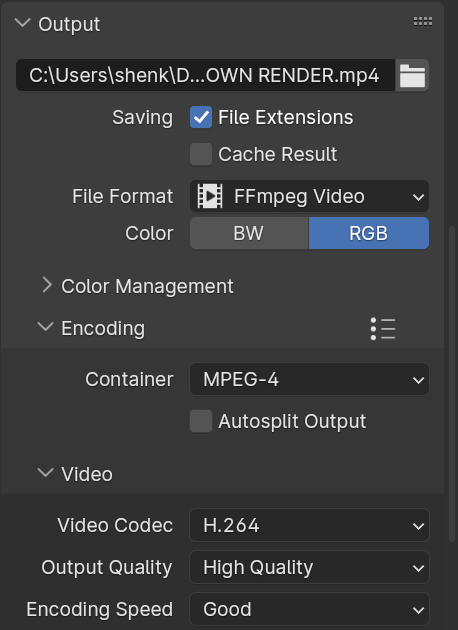


1. This will then output a video file which can be opened and played like any other. These files can also be placed into a video editing software as well for any editing.

**HTML Resource Guide (Supplemental)**

1. The first step for both methods, Blender video rendering and interactive HTML, is to extract the 3D coordinates from the t-SNE, or UMAP, output. In our case the t-SNE was generated using the Rtsne package in R with the file. To save the coordinates to a usable CSV file the code below was executed in R after the t-SNE algorithm has finished.


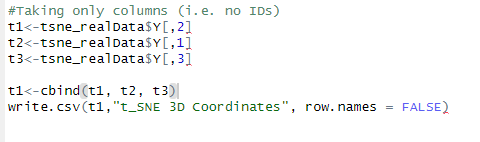


The image above saves the X, Y, and Z coordinates as data objects which are then concatenated together with “cbind” before being written to a usable output with “write.csv.”

1. The second step is to process the coordinates into usable 3D coordinate trios which can be used in both Blender and the HTML files. This task is rather tedious and can be completed by hand using Microsoft Excel or with additional code to help automate the process. The code below (S_1 Code for Converting 3d coordinates to a list (Do not run in Blender).txt) utilizes the CSV output to put the coordinates into the correct format for the downstream processes.


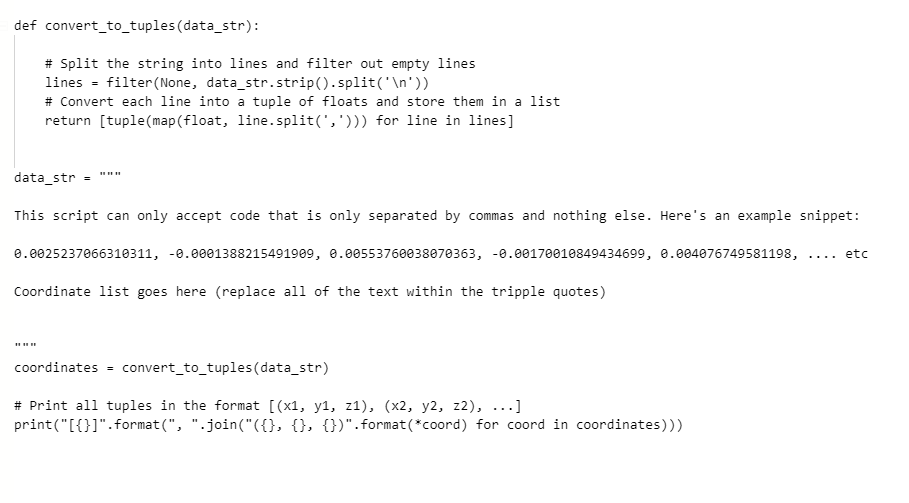


1. To edit the premade file for use with your dataset first download VScode. Then right click the HTML file and then open with VScode. This will then open the HTML file for editing. Alternatively, to begin constructing the html file with VScode from scratch, first open the application and click “new file.” This will open a new text file which will prompt you to choose the file type. You can then click the blue highlighted text and then type “HTML” in the text window at the top of the screen. This will then format the text file for the correct coding language.
2. When using the HTML file provided (S_7 HTML Preset) the primary variables will include position, info, and color. These can all be modified by opening the HTML file with a text editor like VScode. Formatting the required data to match that required for the HTML file can be tedious due to the often large, >1000 datapoints, datasets. In our experience this formatting is best done using an excel file. The excel file included in the resource (S_8 HTML Formatting File) includes five variable columns, xvariable, yvariable, zvariable, infovariable, and colorvariable. These x,y,z variable columns can be replaced with the required coordinates, the infovariable can be replaced with whatever information that is required for hover over information. Although, this could be left empty by removing the text between the apostrophes. The colorvariable is then to be replaced with the desired color’s hexcode. The non-variable columns can then be copy-pasted to match the total number of entries. Then the “concat” function in excel can then be used on these columns (A:M in the example excel) to generate code that can be copy-pasted into the correct section of the HTML file seen below, which begins after the yellow open bracket below.


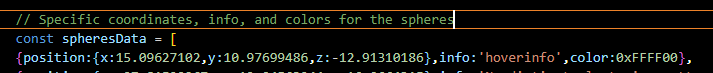


1. Keep in mind that the entries in the sphere data should begin with the first sphere’s curly bracket and end with the last sphere’s ending curly bracket. This means if you copy and paste directly from the excel sheet you may need to remove the comma input after the last sphere.
2. Once the HTML file has had the desired variables added simply save the file, ensuring it is kept as in HTML format. Then simply open the file with a double click. This should then render the file in your web browser of choice as an interactive 3D model.


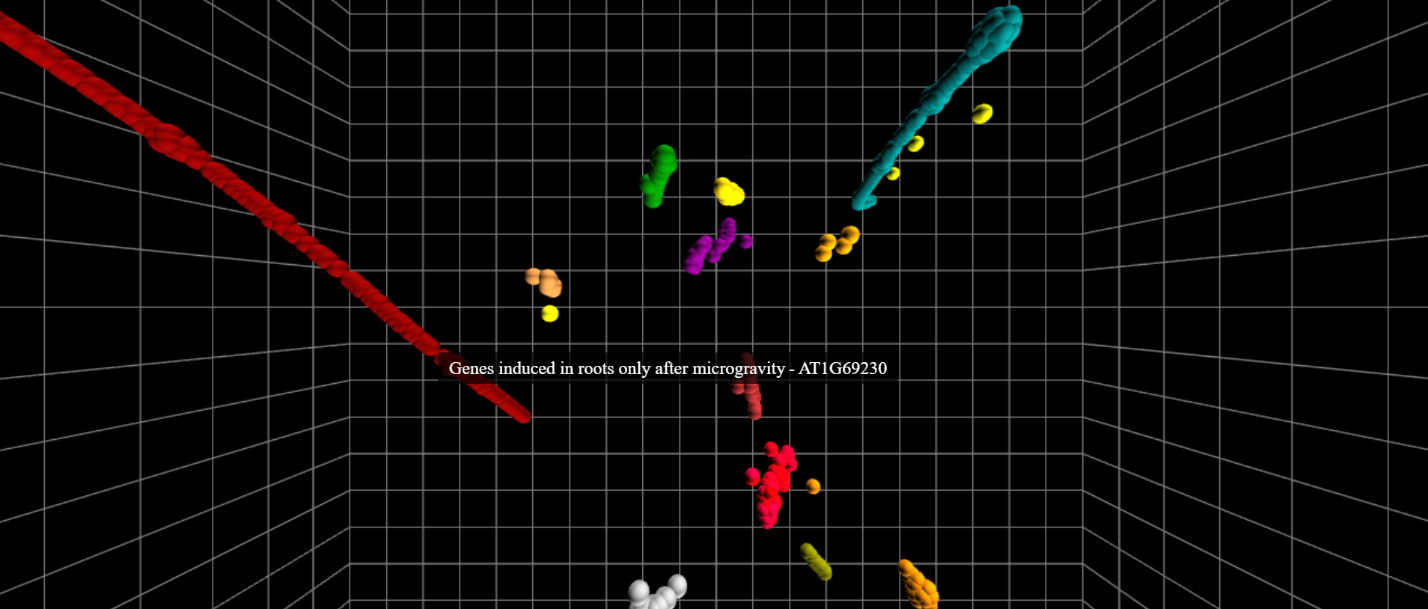

Supplement: Supplementary file 1 — Appendix S1: Supporting Information. [file PPL-177-e70500-s001.zip › ppl70500-sup-0001-Supinfo/S_9 Overall Visualization Guide.docx]
